# Mapping HDX-MS Data to Protein Conformations through Training Ensemble-Based Models

**DOI:** 10.1021/jasms.3c00145

**Published:** 2023-08-07

**Authors:** Ramin E. Salmas, Matthew J. Harris, Antoni J. Borysik

**Affiliations:** Department of Chemistry, Britannia House, King’s College London, London SE1 1DB, U.K.

## Abstract

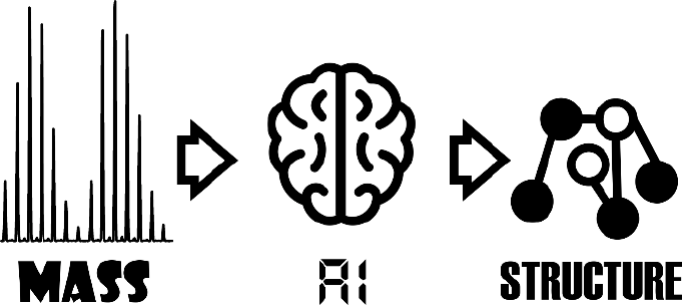

An original approach that adopts machine learning inference to predict protein structural information using hydrogen–deuterium exchange mass spectrometry (HDX-MS) is described. The method exploits an in-house optimization program that increases the resolution of HDX-MS data from peptides to amino acids. A system is trained using Gradient Tree Boosting as a type of machine learning ensemble technique to assign a protein secondary structure. Using limited training data we generate a discriminative model that uses optimized HDX-MS data to predict protein secondary structure with an accuracy of 75%. This research could form the basis for new methods exploiting artificial intelligence to model protein conformations by HDX-MS.

## Introduction

Knowledge of protein structure and the structures of other biomolecules is seen as one of the principal routes to understanding their function. For the increasing list of proteins that thwart classical structural biology, biophysics and simulation can be employed to provide structural models.^1^ Hydrogen–deuterium exchange mass spectrometry (HDX-MS) is an established biophysical technique used to understand protein conformations which has risen in popularity due to recent commercial availability.^2−5^ The approach exploits the natural exchange of covalently bound hydrogen atoms in proteins for deuterium in D_2_O solvent with mass differences between ^1^H and ^2^H permitting the exchange kinetics to be followed by mass spectrometry.^6,7^ Isotope incorporation is typically localized by acid proteolysis which yields a series of partially overlapping peptides of different lengths that can cover the entire protein sequence. In the archetypical HDX-MS experiment isotope incorporation into a protein sample is understood by direct comparison to the HDX behavior of a reference protein.^8^ From these experiments, various effects such as those stemming from point mutations or ligand-binding can be investigated from any accompanying changes in structure and dynamics that alter the kinetics of isotope exchange. One of the main strengths of HDX-MS is that it can uncover changes in protein conformations that are hidden to conventional structural biology.^9−11^ Combined with many other advantages including throughput and sensitivity, HDX-MS has experienced a remarkable increase in popularity over the past decade.

One notable limitation of HDX-MS is the absence of an established approach to exploit the technique for *ab initio* modeling. HDX-MS data are sufficiently rich to accurately identify native structures, but unlike other methods such as NMR or SAXS, HDX is impeded by an incomplete understanding of the structural origins that provoke a particular biophysical output. The pursuit to understand the structural determinants of HDX for protein modeling has given rise to a specialist area covering more than 20 years active research. Over this time, a large number of different paradigms have emerged of varying sophistication and differing interpretations of the structural elements that orchestrate protection from HDX.^12^ One of the most popular approaches for protein modeling by HDX employs the so-called phenomenological method developed by Karplus and co-workers.^13^ Successful modeling of certain structures has been demonstrated using the Karplus method, but the scope of the approach is limited with several examples where it fails to correctly simulate HDX data.^14,15^ A facet shared by virtually all HDX prediction models proposed over the last 2 decades is the use of classical methods to describe HDX.^16−20^ In classical methods, a predictive algorithm linking HDX data to protein conformations must be formulated on expectations of the nature of HDX in contrast to AI where machines are used to create maps between input-output data. Algorithms can be tested and optimized, but classical approaches are ill-equipped at exploring all of the different variables that could potentially influence the HDX characteristics of a particular protein structure.

We describe a method using artificial intelligence (AI) to predict protein structural information by HDX-MS. The application of AI to decipher the relationship between the protein structure and the associated HDX signals is underrepresented in the field despite its obvious relevance to this problem. Our method takes advantage of HDXmodeller an in-house HDX-MS optimizer that models the HDX kinetics of individual amino acids from inputted time-dependent peptide mass changes.^21,22^ The main advantage of HDXmodeller in this application is the accompanying increase in resolution it affords from peptides to amino acids. This resolution change permits structural features to be mapped directly onto individual residues rather than peptides, which limit the model to an average property across each fragment. A model is generated using Gradient Boosting (GB) which is a form of supervised machine learning that utilizes an ensemble of weak predictors akin to decision trees.^23^ Using a limited data set encompassing the HDX-MS profiles of just 5 proteins spanning approximately 500 amino acids, a model is generated capable of predicting residue secondary structure with 75% accuracy. With sufficient training data, other structural features could be mapped to amino acids and described by HDX-MS using this method. A combination of different predictive models obtained by AI could form the basis of new structural modeling techniques based on HDX-MS while also shedding light on the fundamental basis of isotope exchange.

## Materials and Methods

### Materials

Unless stated otherwise, all reagents were purchased from Sigma-Aldrich or Thermo Fisher Scientific. Barnase was produced in-house, and barstar was from Ruth Rose at Queen Mary University of London. Green Fluorescent Protein (GFP) and GFP nanobodies (nb) GFP-nb) and GFP-nbmin were obtained from Rebecca Beavil at King’s College London. All protein samples were diluted to 10–20 μM, aliquoted, and stored at −80 °C prior to use. 1BRS was used for the pdb codes of barnase and barstar with the GFP and GFP-nb structures taken from 3OGO and 3G9A used for the GFP-nbmin structure.

### Methods

#### Hydrogen–Deuterium Exchange Mass Spectrometry

HDX-MS experiments were performed on a Synapt G2Si HDMS in tandem with an Acquity UPLC M-Class system with HDX and automation (Waters Corporation, Manchester, UK) and a LEAP PAL autosampler (Trajan Scientific Europe Ltd., Milton Keynes, UK) for sample management. The mass spectrometer was calibrated against NaI and sample data acquired with lock-mass correction using Leu-enkephalin every 30 s. Data was obtained by diluting 5 μL of protein sample at 10–20 μM into 95 μL of either buffer L (4.5 mM K_2_HPO_4_, 4.5 mM KH_2_PO_4_ in D_2_O, pD 7) or buffer E (4.5 mM K_2_HPO_4_, 4.5 mM KH_2_PO_4_, pH 7) at 20 °C. Data were obtained for 5 different isotope exposure times ranging between 15 s and 4 h, and were collected in triplicate with 6 acquisitions for the reference data. For quenching, 70 μL of each sample was diluted into 70 μL of quench buffer (2.4% formic acid in H_2_O) at 1 °C to reduce further deuteration. Then 50 μL of quenched sample was digested online using a Waters Enzymate BEH pepsin column at 20 °C for 3 min at a flow rate of 200 μL/min in buffer A (H_2_O + 0.1% formic acid, pH 2.5). Peptides were immobilized on a Waters BEH C18 VanGuard precolumn before being separated using a Waters BEH C-18 analytical column with a linear gradient of organic solvent, buffer B (acetonitrile +0.1% formic acid, pH 2.5), from 8 to 40% over 6 min and spectra acquired by electrospray ionization. All trapping and chromatography was performed at 0 °C to minimize the erroneous gain and/or loss of isotope. MS data were acquired for 11 min with the majority of peptides eluting between 2 and 8 min. Clean blanks were taken after each data acquisition which utilizes a gradient of buffer B from 8 to 85% and back over 4 min, repeated twice.

Back exchange controls (BEX) were set up for each protein by loading a single aliquot of protein (<1 mL) into a 3 kDa MWCO Slide-A-Lyzer dialysis cassette followed by dialysis against 100 mL of labeling buffer L overnight at room temperature with gentle stirring. Following the exchange of H_2_O for D_2_O, samples were extracted from the cassette, filtered through a 0.22 μm syringe filter, and incubated at 37 °C for up to 2 weeks. There are several published methods for the production of back exchange control data, also referred to as D_100%_ or D_max_ samples. Published methods normally involve some form of protein denaturation induced by temperature, pH, the addition of chaotropes, or some combination of these methods.^24,25^ Alternative protocols also describe approaches involving the collection and lyophilization of protein digests followed by exposure to D_2_O.^26^ Previous in-house testing involving periodic HDX-MS characterization of protein samples incubated in D_2_O at 37 °C has revealed that most proteins exhibit no further mass increment after a few days of incubation. The additional time provided for the present systems should be sufficient to ensure complete exchange. Additional data were acquired to allow correction for forward exchange (FEX) artifacts. FEX data were obtained by acquiring additional reference data acquisitions but with the quench buffer made using D_2_O to achieve a H_2_O:D_2_O ratio of 1:1 in the final quench of 1:1. BEX data were acquired using the fully exchanged protein samples and treating the sample as a 15 s labeled aquation. In this way, each experimental peptide had unique control data with an identical polypeptide sequence, and all control data were obtained in triplicate. Following data acquisition, reference data sets were initially analyzed using the ProteinLynx Global Server (PLGS) v3.0.2 (Waters Corporation, Manchester, UK) software and the associated ion accounting generated. HDX-MS data were then analyzed using DynamX v3.0.0 (Waters Corporation, Manchester, UK) and the relative fractional uptake (RFU) of each peptide subsequently determined from the centroid masses of each spectral envelope. All RFUs were then corrected for FEX and BEX artifacts according to the following expression, where RFU_corr_, RFU_exp_, RFU_FEX_, and RFU_BEX_ are the respective corrected, experimental, forward, and back exchange RFU for each labeling time point (eq 1).
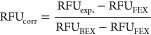
1

#### HDX-MS Data Optimization

The corrected RFU data for each protein were submitted to HDXmodeller for optimization to model the HDX exchange rates (*k*_obs_) of each amino acid (https://hdxsite.nms.kcl.ac.uk/Modeller). An output file containing the intrinsic exchange rates (*k*_int_) of each amino acid was first generated using the online tool k-intrinsic (https://hdxsite.nms.kcl.ac.uk/kintrinsic) with a temperature setting of 293.15 K and a pD of 7.0. Residue resolved protection factors (PFs) were then obtained by uploading the corrected RFU data along with the *k*_int_ file for each protein and the data optimized using the default settings. PFs are outputted by HDXmodeller and calculated from the ratio of *k*_obs_ to *k*_int_ expressed as the natural logarithm of the PF value (ln *P*). HDXmodeller utilizes a bespoke validation method that images the error surface following optimization and then uses this information to quantify the degree of certainty in the model data using a R-matrix value which is any number between 0 and 1. To increase the utility of the R-matrix scores, the experimental data for each protein was subdivided into different subsections and optimized separately. This strategy was adopted because of the variable success rate of optimization across any HDX-MS data set. The preparation of subsections allowed multiple validation outputs to be generated across each protein sequence rather than a single R-matrix score for each protein which would have little utility. The generation of data subsections was facilitated by deleting a limited number of peptides with the identification of peptides suitable for deletion guided by the Occupier tool of HDXsite which can identify weakly constrained peptides and the peptide error scores outputted by HDXmodeller.^21,22^ Data subsection preparation was an iterative process guided by the R-matrix score to maximize the number of subsections generated with minimal loss in the ability of the data to constrain the optimization. It is instructive to note that peptide deletion generally affected redundancy not coverage, and as such, this process had virtually no impact on the number of residues used for model development. Overall 26 different subsections of HDX-MS data were prepared from 5 different proteins with model exchange rates calculated for approximately 500 amino acids (Table 1). All of the experimental data used with this research is downloadable from the HDXmodeller web site.

**Table 1:** Summary of R-matrix scores for the 5 proteins and data subsections. The start and end amino acids for each subsection are indicated, along with the associated R-matrix scores in parentheses. The overall R-matrix score following HDX-MS data optimization of each protein without subdivision of the data is also provided

protein	barnase	barstar	GFP	GFP-nb	GFP-nbmin
overall R-matrix	(0.775)	(0.726)	(0.671)	(0.645)	(0.795)
subsection 1	A1-Y13 (0.924)	E8-L16 (0.362)	L7-F46 (0.760)	L5-L21 (0.832)	A2-L22 (0.521)
subsection 2	Y13-A43 (0.699)	L16-L34 (0.892)	F46-F99 (0.594)	S22-W37 (0.272)	S23-E48 (0.871)
subsection 3	A43-F56 (0.421)	L34-E52 (0.594)	F100-F130 (0.588)	E48-F69 (0.560)	E48-T70 (0.838)
subsection 4	F56-D93 (0.891)	E52-L71 (0.360)	F130-F165 (0.702)	L82-Y95 (0.218)	T70-C97 (0.707)
subsection 5	W94-I109 (0.635)	Q72-T85 (0.306)	K166-L207 (0.661)	Y95–F103 (0.722)	D121-H139 (0.745)
subsection 6			L207-T230 (0.606)		

#### Machine Learning

500 data points constituting different amino acids over 5 proteins and 4 features including *k*_obs_, *k*_int_, R-matrix, and amino acid type were used as training data. The amino acid type included structural features for each amino acid with the names encoded between 0 and n classes -1 in order to transform the categorical scale to numerical predictors. Crystal structures of the relevant proteins were taken and used to determine the phi and psi dihedral angles of each amino acid which were then binned into binary classes using kernel density classification such that every amino acid was defined as either β-strand or α-helix. A GB algorithm using the scikit-learn Python library was then applied to learn how to map the input features to amino acid secondary structure.^27^ GB is a type of supervised machine learning that generates a series of decision trees, with each new tree attempting to improve the error. The procedure of building trees and minimization continues until the model is either overfit or there is no change in the residuals with a differentiable loss function used to improve the output in each tree. The data included in GB are the input variables and the output classification (eq 2), where *x*_*i*_ and *y*_*i*_ refer to the variables and binary classification targets respectively.
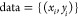
2

A single leaf was initially built and given a value based on the log(odds) of class 1, for example, the probability of each amino acid being a β-strand. This value, which is the initial prediction, was then transformed into a probability prediction using a logistic function by softmax. Differences between the actual and predicted values were then quantified by a loss function (*L*) defined in the compact form as follows (eq 3).
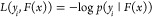
3

An initial model was generated using constant values as follows, where *x* refers to the input values (*k*_obs_, *k*_int_, R-matrix value, and amino acid), *y*_*i*_ is the observed classification value (0 or 1) of each data point (i), and γ is the log(odds). Argmin denotes the process of searching for optimum values of γ to minimize the loss function (eq 4).
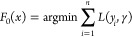
4

A cross entropy loss function, which is differentiable with respect to the predicted value or log(odds), was then utilized in the gradient descent algorithm in order to identify the correct direction to be followed with changes in γ.^28^ The cross-entropy or logarithmic loss function is based on predicted probability and is defined as follows: where *y*_*i*_ is the target variable (0 or 1) and *p* is the predicted probability of class 1 in decision trees in each iteration (eq 5).
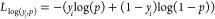
5

Transformation of the loss function was performed, allowing it to function using predicted log(odds) rather than probability according to the following relationship (eq 6).
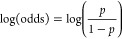
6

The loss function was then converted into a function of log(odds) (eq 7) with *p* redefined to log(odds) using the softmax transformation (eq 8).
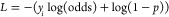
7
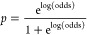
8

The loss function expression can be simplified as follows to show the log(odds) for different outputs for the individual leaves (eqs 9, 10).
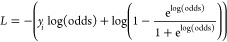
9
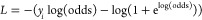
10

Since this loss function is differentiable, the derivative can be taken with respect to the log(odds) (eq 11).
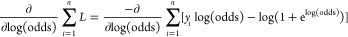
11

The derivate of the first part (*y*_*i*_ log(odds)) of eq 10 is the negative of the observed value, and for the derivate of the second part (−log(1 + e^log(odds)^) the chain rule was used (eqs 12, 13), and this was solved by setting the value to 0 so that *p* was equal to the mean of *y* (eq 14).
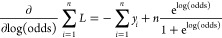
12
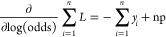
13
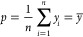
14

Pseudoresiduals (*r*) were calculated following each iteration and compared to the previous prediction using the derivative of the loss function as shown, where *m* denotes the index of each tree and *i* is the number assigned to each data point (eq 15).
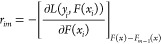
15

This can be simplified to show the deference between the observed data (*y*_*i*_) and predicted probability (*p*) (eq 16).
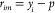
16

Following this, a regression tree was fitted to the residual values to generate terminal regions. The output value (γ) for each leaf was then calculated as shown where *i* and *j* refer to the leaf number, and the total number of the leaves, respectively (eq 17).
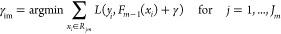
17

The loss function was then added, and the second order Taylor polynomial was employed to simplify the function as follows, where γ is the output for each leaf (*i*) on each tree (*m*) (eq 18).
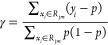
18

A search then occurs on individual trees to optimize values of γ with the objective of improving the output of the new function (*F*_*m*_) with respect to the previous prediction (*F*_*m*–1_) expressed as follows, where v is the learning rate for which a value of 0.2 was used and based on the hyper-parameter optimization (eq 19).
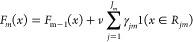
19

#### Model Validation

Validation using domain knowledge is a crucial part in understanding the quality of a model generated by GB. In instances where the size of the training data is limited, setting aside 20% of the data for the purpose of validation can be inefficient in determining the overall quality of the model. In these cases the optimum solution is to adopt a cross validation (CV) strategy in which the training set is split into K folds (*k*∈ {1,..., K}) and the system is trained and validated by the *k*th data set interchangeably akin to a round-robin tournament.^29^ A stratified K-fold validation strategy was implemented using a scikit-learn Python Library to evaluate the preference of the model where the whole data was split into 5 (K) parts. The main hyper-parameters in the GB algorithm include the number of boosting stages, learning rate, and number of nodes in each tree which control the type and complexity of the model. Optimal model parameters were found using grid searching with selection based on the loss output. Three evaluation strategies we utilized to measure and visualize the performance of the algorithm involving confusion matrices, calibration curves, and receiver operating characteristic (ROC) plots. The confusion matrix provided a visual means to evaluate the accuracy of the model in the testing data set by indicating the percentage of true positives (TP), false positives (FP), true negative (TN), and false negative (FN) cases of the label classes. Confusion matrices consider the label class for the predicted and observed values with the calculation based on the mismatch between the binary labels. Calibration curves were utilized to measure the difference between the actual values and the predicted probabilities and understand how the model was corroborated.^30^ Calibration curves provide more confidence for the prediction of the algorithm from a probabilistic perspective where a more constant baseline could be interpreted as a more calibrated model. The squared error of the predictive probabilities compared to actual class data was calculated using Brier Score with the Brier Skill Score used to understand how the model improved compared to previous models.^31^ A ROC plot was finally used to provide information about the relationship between the true positive rate (TPR) and the false positive rate (FPR) at different threshold values. The plot provided a visual representation of the diagnostic ability of the model with the area under the curve (AUC) yielding a quantitative measure of the ability of the algorithm to discriminate the classes. The AUC describes the probability of correctly classifying examples of each class taken a random. A value of 0.5 is equivalent to random chance, with the model providing no benefit beyond a coin flip. An AUC of 1.0 represents a perfectly accurate model that is able to discriminate each class without error.

## Results

**Figure 1:**
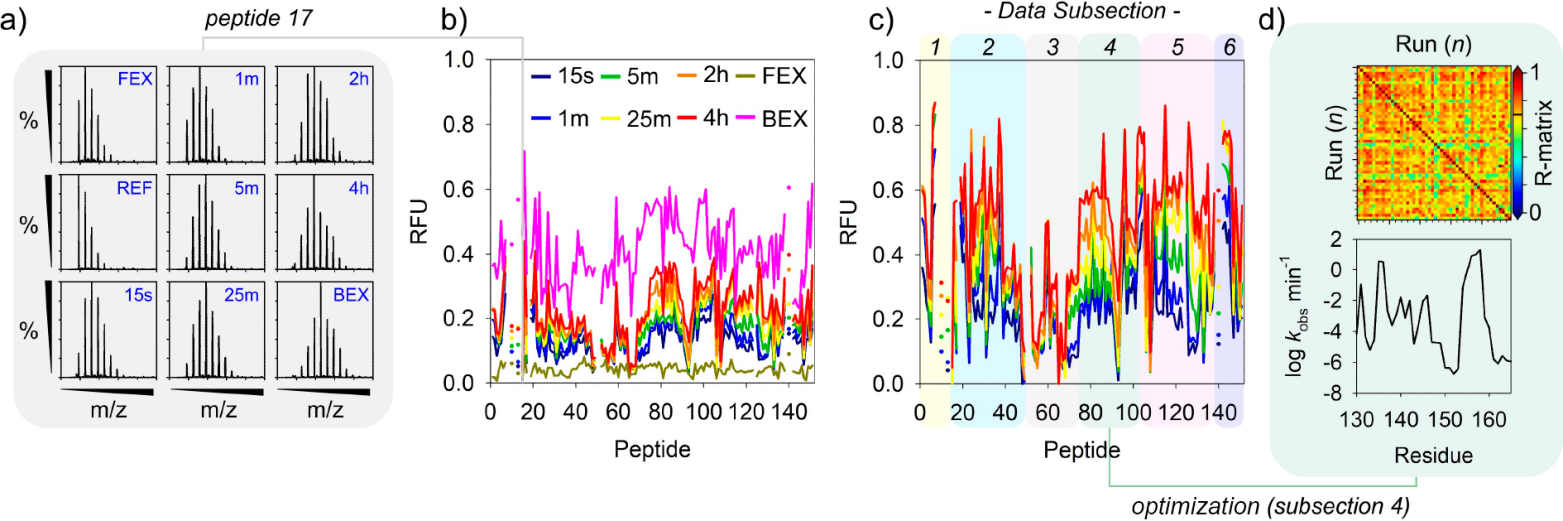
Overview of HDX-MS data acquisition and processing. (a) Example HDX-MS spectra for green fluorescent protein (GFP). Representative spectra are shown for 6 different isotope labeling times as well as the unlabeled reference spectrum (REF) and spectra for back (BEX) and forward exchange (FEX) control data. The *m*/*z* scale is between 569 and 574 throughout. (b) Overview of peptide mass changes for GFP reported as relative fractional uptake (RFU). RFUs are shown for 6 different isotope exposure times along with the back and forward exchange RFUs. Missing data in the profile are due to peptide removal to aid in the creation of unique data subsections or represent peptides removed due to high error. (c) Same data as for (b) but with the RFU corrected for back and forward exchange. Peptide removal allowed the generation of 6 different data subsections as shown. (d) Each subsection of HDX-MS data was submitted for optimization in turn using HDXmodeller. Following optimization the main outputs were the residue resolved HDX exchange rates (*k*_obs_) and the R-matrix validation score for each subsection which is taken as the arithmetic mean of the pairwise correlation coefficients between all replicate optimization runs. These data were then used as input for development of the gradient boosting algorithm.

HDX-MS data were acquired for 5 different proteins including barnase, barstar, green fluorescent protein (GFP) and 2 GFP-binding nanobodies (nb) GFP-nb and GFP-nbmin. Since the purpose of this research was to assess the feasibility of using AI to define a predictive structural algorithm based on HDX-MS data, no particular emphasis was placed on protein selection. The only real constraint was that the data should include back and forward exchange values for each peptide to facilitate RFU correction. Successful method development is viewed as the first step in establishing a foundation for future models that are more highly tuned to specific structural characteristics. Data were acquired over 6 different isotope exposure times along with data for reference samples and control data, allowing correction for back and forward exchange artifacts. Raw HDX-MS data were processed, and the corrected relative fractional uptake (RFU) was calculated for each peptide and labeling time point (Materials and Methods). Corrected RFU were then submitted for data optimization by HDXmodeller to extract the underlying exchange kinetics and enable residue resolution. Rather than submitting individual optimization runs for each protein, each data set was first subdivided into separate subsections each of which was then optimized separately. HDXmodeller provides a validation output for each optimization based on a unique R-matrix score taken as the arithmetic mean of the pairwise correlation coefficients (*R*) of all optimization replicates in a production run. The R-matrix in a bespoke validation method for HDX-MS data optimization provides a score from 0 to 1 that quantifies the quality of the constraints and is highly correlated with the accuracy of modeled data. Optimisation of individual subsections allows the generation of multiple R-matrix scores for each data set, thereby increasing the detail at which the validation outputs are reported (Materials and Methods). Overall 26 different subsections of data were optimized for the 5 proteins encompassing 500 amino acids (Table 1). The R-matrix scores varied from poor (0.218) to excellent (0.924), and the intention was to allow the AI to utilize these scores in model development (Figure 1).

**Figure 2:**
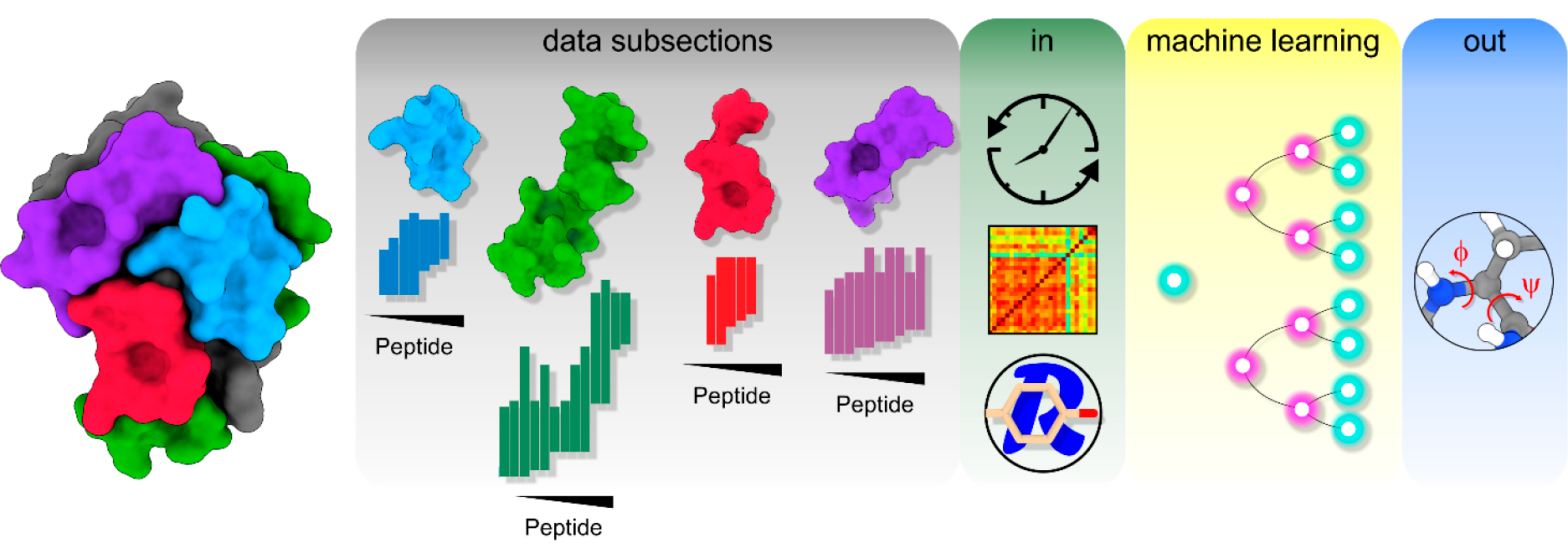
Example workflow of supervised learning using Gradient Boost. Left to right: HDX-MS data of each protein was subdivided into different subsections and each subsection submitted separately for optimization by HDXmodeller. Example data subsections and their associated peptide maps are shown. Optimized data outputs including the observed exchange rates (*k*_obs_), the R-matrix scores calculated from the images of covariance matrices and the amino acid type were used as inputs (in) for supervised machine leaning. Each amino acid was labeled with the input values along with a one-dimensional target for amino acid secondary structure type taken from the dihedral angles (out). A model was then trained using a GB algorithm to predict the secondary structure from the HDX-MS input data.

The entire data set, involving approximately 500 amino acids, was then used to train a model using GB, a type of supervised machine learning that implements an ensemble of many weak learners or decision trees designed to gradually improve the mismatch between the predicted and true values. In order to search for the best input-output mapping algorithm employed by the machine for learning from the data set, diverse machine learning models with different integrated mapping methods were built and evaluated including Nearest Neighbors, Linear Support Vector Machines (SVM), Radial Basis Function (RBF) SVM, Gaussian Process, Decision Tree, Random Forest, Multilayer Perceptron (MLP), AdaBoost, Naive Bayes, Quadratic Discriminant Analysis (QDA), Histogram-based GB, Extra Trees, LightGBM, Logistic Regression, and XGBoost. The same strategy based on generating confusion matrices, calibration plots, and ROC AUC scores for the validation data set was applied for measuring the uncertainty existing in the predictions of the models. The comparison between the certainty in the models, demonstrated that the performance of these methods was inferior to GB for this specific classification task (Figure S1). Each amino acid in the model consisted of a four-dimensional covariate of *k*_obs_, *k*_int_, R-matrix, and amino acid type along with a one-dimensional target for secondary structure. Assignment of amino acids in binary classes of β-sheet or α-helix was performed by kernel density classification on the basis of their phi and psi dihedral angles taken from the associated pdb files. No imbalance between the 2 classes was observed with the binary classifications sharing almost equal proportions of β-sheet and α-helical conformations. The many hyper-parameters that define the structure of the GB model including the number of trees and internal nodes, type of loss function and learning rate were defined through several rounds of optimization to maximize model accuracy (Figure 2, Materials and Methods).

**Figure 3:**
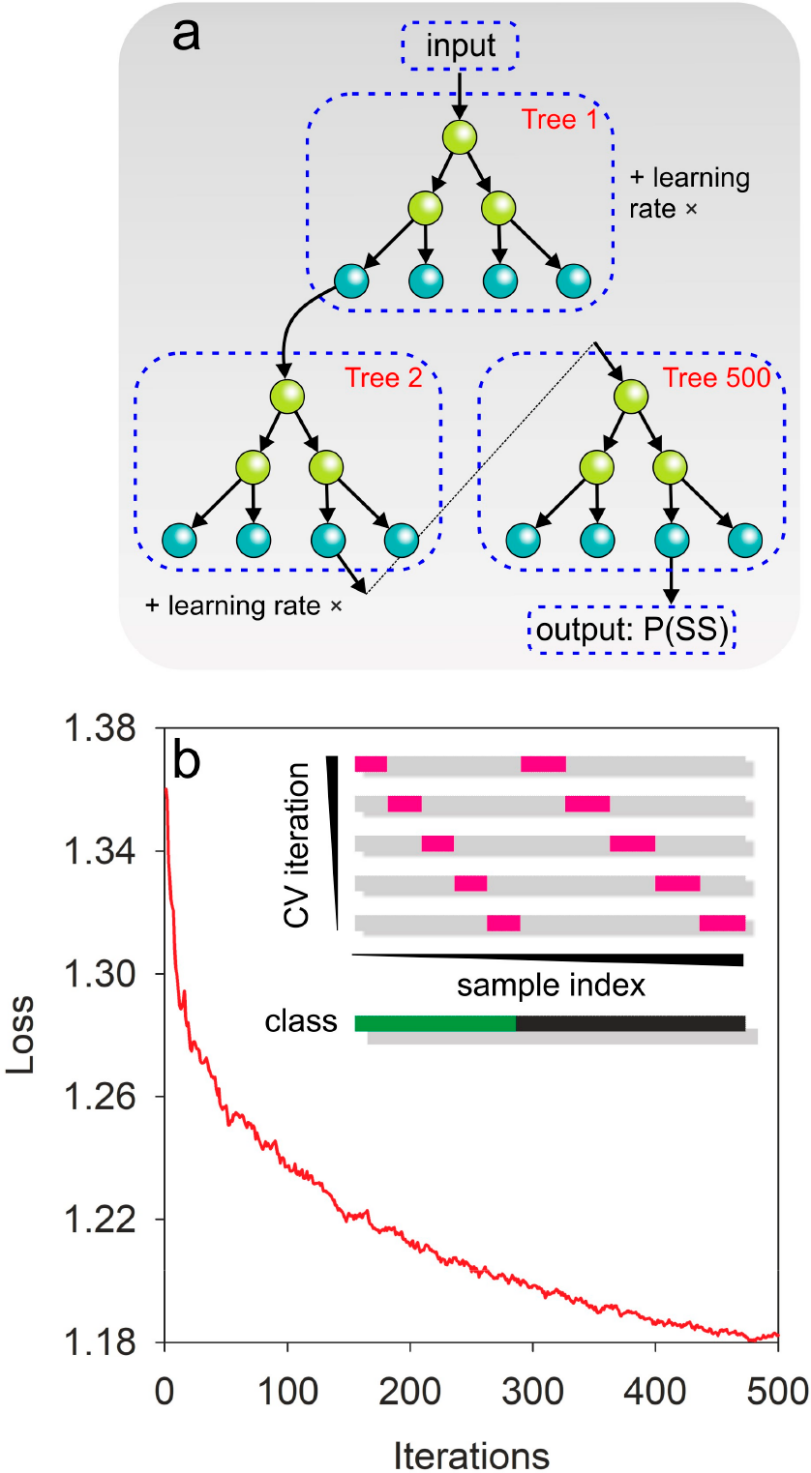
Organization of the decision trees and model validation. (a) Representation of the structure of the GB algorithm to determine the pobability (P) of secondary structure (SS). GB utilizes an ensemble of weak learners or decision trees where leaf nodes (blue) of one tree are inputs to the root nodes (green) of the next tree. The leaf nodes of the last decision tree will be the best and final output of the model as determined by the error. The learning rate or shrinkage factor is used to slow down the contribution of trees in each stage and avoid overfitting of the training data. (b) Model error (loss) for each iteration and cross validation (CV) plot depicting model training and validation (insert). Training and validation was iterated 5 times with 80% of the data allocated to training (gray) and 20% allocated to validation (pink) in each iteration. Note how the indices of the data used shift across in each step to cover 100% of the data. Classes were balanced with nearly identical proportions of data for β-sheet (green) and α-helix (black) targets.

The quality of the model was then evaluated using cross validation (CV) in which 20% of the data was used to evaluate the performance of a model trained by the remaining 80%. During CV both the training and validating processes were conducted 5 times using entirely different data constituting the training and evaluation data in each cycle. This validation strategy was employed because of the increased reliability of CV in assessing model quality arising from its capacity to use the entire data set for validation rather than simpler methods that only utilize the highest quality data. The training process was carried out over 500 iterations during which the different hyper-parameters were optimized. Gradient descent was used to follow the error during optimization and indicate the point at which the model had converged (Figure 3, Materials and Methods).

**Figure 4:**
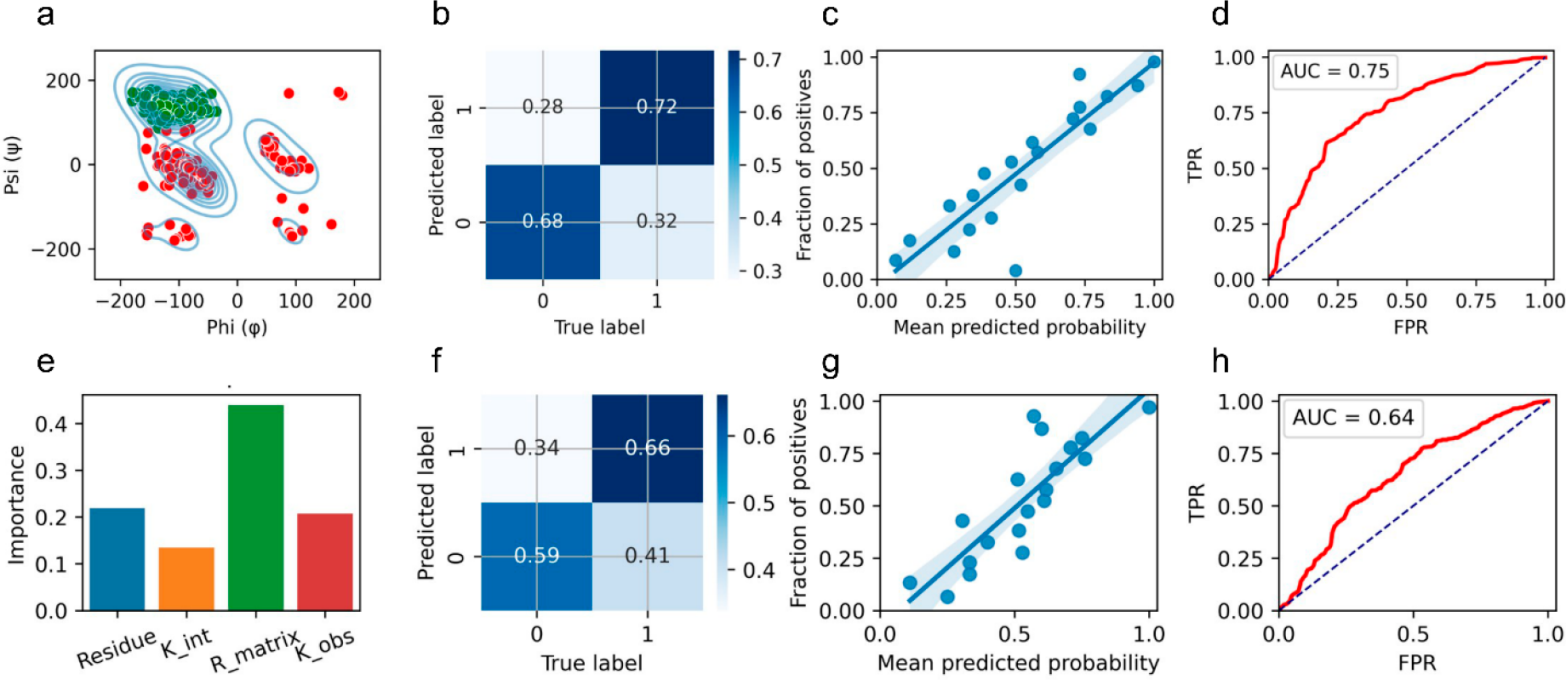
(a) Ramachandran plot showing the binary classification of amino acids corresponding to β-sheet (green) and α-helical conformations (red). (b) Confusion matrix of the testing data set shown as a heat map with the normalized number of true positive and true negative classes being 0.68 and 0.72, respectively. (c) Calibration curve of the relationship between predicted probability classes and the true classes binned into 20 random groups. (d) ROC plot for the observed and predicted probability classes for the testing data set separated from the whole data set using cross validation; the accuracy of the model taken from the area under the curve (AUC) is 0.75. (e) Feature importance plot for the 4 input parameters of the model with the R-matrix score having the most important role in classifying the data. (f–h) Data are shown as for (b–d) but with omission of R-matrix from the input labels; exclusion of the R-matrix score reduces the AUC 0.64.

The extent to which the model was able to discriminate between secondary structure types was then evaluated. A confusion matrix was initially printed, which reported the percentage of correctly identified negative labels at 68% and the percentage of correctly identified positive labels at 72%. Further insight into model accuracy was then obtained through the preparation of a calibration curve based on the true label classes and the probability of the respective classes. The GB method is able to provide estimates of class probabilities that can be interpreted as a confidence factor in the calibration curve. Preparation of the calibration curves initially involved random partitioning of the data into 20 different bins. For each of the binned data sets, the fraction of positive cases was then determined and compared to the mean predicted probability of correct secondary structure assignment. The calibration curve indicated a robust model with a corresponding brier score (BS) loss of 0.20, with the BS indicating model linearity reported by a value between 0 and 1 with lower values being optimal. A ROC curve was then plotted to provide a visual representation of the true positive rate (TPR) against the false positive rate (FPR) determined across a wide range of thresholds. From the ROC plot the area under the curve (AUC) was also calculated revealing a model accuracy of 75% (Figure 4).

The importance of different input features in the model was then investigated. Of the 4 inputs, *k*_int_ was found to have the smallest effect on the predictive model, which is expected given that *k*_int_ depends on variables that are unrelated to higher order structure. Amino acid type and *k*_obs_ had similar contributions to the model, and while there are known secondary structure propensities for certain amino acids the training data is likely too small to detect and model these trends. The R-matrix validation score was unexpectedly found to have the greatest contribution to model accuracy. To confirm the importance of R-matrix, data were reoptimized but with the omission of this term which resulted in a considerable decrease in model accuracy to 64% with a corresponding reduction in the BS from 0.20 to 0.234 (Figure 4). While the relationship of R-matrix with secondary structure is unknown, it is apparently exploited by the model for the characterization of *k*_obs_. This suggests that the R-matrix term may have an important role in AI applications that rely on optimized HDX-MS data for model generation.

## Conclusion

Leveraging HDX-MS for protein modeling has transformative potential across biosciences owing to the many advantages of the technique and its applicability to a diverse range of protein systems. While recent advances in computational methods has seen a step change in the accuracy of *ab inito* protein structures, the reliability of these models can really only be assessed by experiment.^32^ More tools are therefore required capable of exploiting biophysics for the structural characterization of biomolecules. Focusing on this critical aspect of structural biology, we have described a method based on GB capable of predicting protein secondary structure from optimized HDX-MS data. The development of predictive algorithms for HDX is complex, and despite a plethora of models being proposed over the last 2 decades, methods to determine protein conformations by HDX-MS have yet to be established. The ability of AI to effectively learn from data experiences and find a map between features and responses that may not be apparent with classical methods makes it highly suited for this application. In spite of the relevance of AI for linking structural features to HDX, the use of the technology in this area has been largely ignored. A knowledge-based predictor of HDX protection factors is the only other known example, but this approach did not utilize experimental data and was developed to make predictions directly from sequence.^33^ Our model clearly demonstrates the feasibility of using AI to develop algorithms capable of predicting structures using HDX. Direct application of this method was not pursued because of the spacity of the training data and low expectations of the diagnostic power of models that can classify only secondary structure. However, our method defines a roadmap for the development of more sophisticated models with potential for direct application as more training data becomes available.

The use of optimized HDX-MS data in this research had an important role in model development as it allowed structural features to be mapped directly onto amino acids. This is not possible with conventional HDX-MS data, which is resolved at the peptide level and therefore represents a one-dimensional input poorly suited for this type of classification problem. In AI methods the most efficient strategy describing tasks that involve chemical structures with high complexity is to map multidimensional presentations of data to responses or targets, and in most cases one-dimensional vectors conveying less informative data cannot be classified in this way. The utilization of HDXmodeller to optimize the HDX-MS data is therefore appropriate in this research as it increased the number of classifiable features which benefits nonlinear models. Nevertheless, the projection of high-resolution HDX-MS data is a nontrivial optimization problem prone to errors with highly deterministic outputs that depend heavily on the initial guess values. To overcome this problem our model exploited a bespoke R-matrix term which is a value assigned postoptimization and related to the accuracy of model data. Interestingly, the R-matrix value was found to be the most important feature in model development despite having an unknown connection to protein secondary structure in the model. This unique validation parameter may therefore have a vital role in the development of future AI models utilizing HDX-MS. A limitation of the present model regards the handling of coil structure, which has been overlooked. Since the model is based on dihedral angles, it is not well suited for the prediction of coils given the large range of dihedrals these structures can adopt. The presence of coils in the training set may also be a source of misclassification, reducing model accuracy, and a different approach would be needed to map coil structure to HDX-MS data. Rationalization of these results at the fundamental level is challenging due to the low interpretability of black-box models such as those generated by GB. Nevertheless, the capacity to assign protein secondary structure using HDX-MS data implies some form of relationship between isotope exchange and different classes of secondary structure. β-Sheets may be less protected overall because of their lower stability, increased flexibility, and greater exposure of backbone NH groups relative to α-helices.^34−37^ However, the ability of HDX to discriminate between α and β secondary structures may be a result of some unknown conformational features. It will be interesting to follow the emergence of any future relationships uncovered by AI on the fundamental relationship between HDX and protein conformations.

## Data Availability

The codes for the gradient boosting algorithm can be obtained from GitHub https://github.com/raminsalmas/HDX_Folding. All experimental data used with this research in the form of the corrected RFU for each peptide can be downloaded from the “example” resource accessed from the HDXmodeller web page https://hdxsite.nms.kcl.ac.uk/Modeller.

## Supporting Information Available

The following Supporting Information is available free of charge at the ACS Web site Figure S1. Caption () The Supporting Information is available free of charge at https://pubs.acs.org/doi/10.1021/jasms.3c00145.

Ability of different types of machine learning algorithms to assign protein secondary structure by HDX-MS (PDF)

## References

[ref1] Kim S. J.; Fernandez-Martinez J.; Nudelman I.; Shi Y.; Zhang W.; Raveh B.; Herricks T.; Slaughter B. D.; Hogan J. A.; Upla P.; Chemmama I. E.; Pellarin R.; Echeverria I.; Shivaraju M.; Chaudhury A. S.; Wang J.; Williams R.; Unruh J. R.; Greenberg C. H.; Jacobs E. Y.; Yu Z.; de la Cruz M. J.; Mironska R.; Stokes D. L.; Aitchison J. D.; Jarrold M. F.; Gerton J. L.; Ludtke S. J.; Akey C. W.; Chait B. T.; Sali A.; Rout M. P. Integrative structure and functional anatomy of a nuclear pore complex. Nature 2018, 555 (7697), 475–482. 10.1038/nature26003. 29539637 PMC6022767

[ref2] Englander S. W.; Kallenbach N. R. Hydrogen exchange and structural dynamics of proteins and nucleic acids. Q. Rev. Biophys. 1983, 16 (4), 521–655. 10.1017/S0033583500005217. 6204354

[ref3] Narang D.; Lento C.; Wilson D. J. HDX-MS: An Analytical Tool to Capture Protein Motion in Action. Biomedicines 2020, 8 (7), 224–244. 10.3390/biomedicines8070224. 32709043 PMC7399943

[ref4] Zheng J.; Strutzenberg T.; Pascal B. D.; Griffin P. R. Protein dynamics and conformational changes explored by hydrogen/deuterium exchange mass spectrometry. Curr. Opin Struct Biol. 2019, 58, 305–313. 10.1016/j.sbi.2019.06.007. 31351767

[ref5] Engen J. R.; Botzanowski T.; Peterle D.; Georgescauld F.; Wales T. E. Developments in Hydrogen/Deuterium Exchange Mass Spectrometry. Anal. Chem. 2021, 93 (1), 567–582. 10.1021/acs.analchem.0c04281. 33112590

[ref6] Bai Y.; Milne J. S.; Mayne L.; Englander S. W. Primary structure effects on peptide group hydrogen exchange. Proteins 1993, 17 (1), 75–86. 10.1002/prot.340170110. 8234246 PMC3438223

[ref7] Zhang Z.; Smith D. L. Determination of amide hydrogen exchange by mass spectrometry: a new tool for protein structure elucidation. Protein Sci. 1993, 2 (4), 522–31. 10.1002/pro.5560020404. 8390883 PMC2142359

[ref8] Houde D.; Berkowitz S. A.; Engen J. R. The utility of hydrogen/deuterium exchange mass spectrometry in biopharmaceutical comparability studies. J. Pharm. Sci. 2011, 100 (6), 2071–86. 10.1002/jps.22432. 21491437 PMC3164548

[ref9] Canet D.; Last A. M.; Tito P.; Sunde M.; Spencer A.; Archer D. B.; Redfield C.; Robinson C. V.; Dobson C. M. Local cooperativity in the unfolding of an amyloidogenic variant of human lysozyme. Nat. Struct. Biol. 2002, 9 (4), 308–15. 10.1038/nsb768. 11887182

[ref10] Choi J. H.; Banks A. S.; Estall J. L.; Kajimura S.; Boström P.; Laznik D.; Ruas J. L.; Chalmers M. J.; Kamenecka T. M.; Blüher M.; Griffin P. R.; Spiegelman B. M. Anti-diabetic drugs inhibit obesity-linked phosphorylation of PPARgamma by Cdk5. Nature 2010, 466 (7305), 451–6. 10.1038/nature09291. 20651683 PMC2987584

[ref11] Ahn M.; Hagan C. L.; Bernardo-Gancedo A.; De Genst E.; Newby F. N.; Christodoulou J.; Dhulesia A.; Dumoulin M.; Robinson C. V.; Dobson C. M.; Kumita J. R. The Significance of the Location of Mutations for the Native-State Dynamics of Human Lysozyme. Biophys. J. 2016, 111 (11), 2358–2367. 10.1016/j.bpj.2016.10.028. 27926837 PMC5153563

[ref12] Devaurs D.; Antunes D. A.; Borysik A. J. Computational Modeling of Molecular Structures Guided by Hydrogen-Exchange Data. J. Am. Soc. Mass Spectrom. 2022, 33 (2), 215–237. 10.1021/jasms.1c00328. 35077179

[ref13] Vendruscolo M.; Paci E.; Dobson C. M.; Karplus M. Rare Fluctuations of Native Proteins Sampled by Equilibrium Hydrogen Exchange. J. Am. Chem. Soc. 2003, 125 (51), 15686–15687. 10.1021/ja036523z. 14677926

[ref14] Skinner J. J.; Lim W. K.; Bédard S.; Black B. E.; Englander S. W. Protein hydrogen exchange: Testing current models. Protein Sci. 2012, 21 (7), 987–995. 10.1002/pro.2082. 22544567 PMC3403436

[ref15] Harris M. J.; Raghavan D.; Borysik A. J. Quantitative Evaluation of Native Protein Folds and Assemblies by Hydrogen Deuterium Exchange Mass Spectrometry (HDX-MS). J. Am. Soc. Mass Spectrom. 2019, 30 (1), 58–66. 10.1007/s13361-018-2070-3. 30280315 PMC6318237

[ref16] Hilser V. J.; Freire E. Structure-based calculation of the equilibrium folding pathway of proteins. Correlation with hydrogen exchange protection factors. J. Mol. Biol. 1996, 262 (5), 756–72. 10.1006/jmbi.1996.0550. 8876652

[ref17] de Vries S. J.; van Dijk M.; Bonvin A. M. The HADDOCK web server for data-driven biomolecular docking. Nat. Protoc 2010, 5 (5), 883–97. 10.1038/nprot.2010.32. 20431534

[ref18] Craig P. O.; Lätzer J.; Weinkam P.; Hoffman R. M.; Ferreiro D. U.; Komives E. A.; Wolynes P. G. Prediction of native-state hydrogen exchange from perfectly funneled energy landscapes. J. Am. Chem. Soc. 2011, 133 (43), 17463–72. 10.1021/ja207506z. 21913704 PMC3203634

[ref19] Marzolf D. R.; Seffernick J. T.; Lindert S. Protein Structure Prediction from NMR Hydrogen-Deuterium Exchange Data. J. Chem. Theory Comput. 2021, 17, 2619–2629. 10.1021/acs.jctc.1c00077. 33780620

[ref20] Liu T.; Pantazatos D.; Li S.; Hamuro Y.; Hilser V. J.; Woods V. L. Quantitative assessment of protein structural models by comparison of H/D exchange MS data with exchange behavior accurately predicted by DXCOREX. J. Am. Soc. Mass Spectrom. 2012, 23, 43–56. 10.1007/s13361-011-0267-9. 22012689 PMC3889642

[ref21] Salmas R. E.; Borysik A. J. HDXmodeller: an online webserver for high-resolution HDX-MS with auto-validation. Communications Biology 2021, 4 (1), 199 10.1038/s42003-021-01709-x. 33589746 PMC7884430

[ref22] Salmas R. E.; Borysik A. J. Characterization and Management of Noise in HDX-MS Data Modeling. Anal. Chem. 2021, 93 (19), 7323–7331. 10.1021/acs.analchem.1c00894. 33961396

[ref23] Friedman J. H. Stochastic gradient boosting. Computational Statistics & Data Analysis 2002, 38 (4), 367–378. 10.1016/S0167-9473(01)00065-2.

[ref24] Mayne L. Hydrogen Exchange Mass Spectrometry. In Methods in Enzymology; Kelman Z., Ed.; Academic Press: 2016; Vol. 566, Ch 13, pp 335–356. 10.1016/bs.mie.2015.06.035 PMC5858910 26791986

[ref25] Yan X.; Maier C. S. Hydrogen/Deuterium Exchange Mass Spectrometry. In Mass Spectrometry of Proteins and Peptides: Methods and Protocols; Lipton M. S., Paša-Tolic L., Eds.; Humana Press: Totowa, NJ, 2009; pp 255–271.

[ref26] Resing K. A.; Ahn N. G. Deuterium Exchange Mass Spectrometry as a Probe of Protein Kinase Activation. Analysis of Wild-Type and Constitutively Active Mutants of MAP Kinase Kinase-1. Biochemistry 1998, 37 (2), 463–475. 10.1021/bi971750x. 9425067

[ref27] Pedregosa F.; Varoquaux G.; Gramfort A.; Michel V.; Thirion B.; Grisel O.; Blondel M.; Prettenhofer P.; Weiss R.; Dubourg V.; Vanderplas J.; Passos A.; Cournapeau D.; Brucher M.; Perrot M.; Duchesnay É. Scikit-learn: Machine Learning in Python. J. Mach. Learn. Res. 2011, 12, 2825–2830.

[ref28] Ruder S. An overview of gradient descent optimization algorithms. ArXiv(Machine Learning), September 15, 2016, ver.1, 1609.04747.

[ref29] Hastie T.; Tibshirani R.; Friedman J. H.; Friedman J. H. The elements of statistical learning: data mining, inference, and prediction; Springer: 2009; Vol. 2.

[ref30] Kull M.; Silva Filho T. M.; Flach P. Beyond sigmoids: How to obtain well-calibrated probabilities from binary classifiers with beta calibration. Electronic Journal of Statistics 2017, 11 (2), 5052–5080. 10.1214/17-EJS1338SI.

[ref31] Gneiting T.; Raftery A. E. Strictly Proper Scoring Rules, Prediction, and Estimation. Journal of the American Statistical Association 2007, 102 (477), 359–378. 10.1198/016214506000001437.

[ref32] Jumper J.; Evans R.; Pritzel A.; Green T.; Figurnov M.; Ronneberger O.; Tunyasuvunakool K.; Bates R.; Žídek A.; Potapenko A.; Bridgland A.; Meyer C.; Kohl S. A. A.; Ballard A. J.; Cowie A.; Romera-Paredes B.; Nikolov S.; Jain R.; Adler J.; Back T.; Petersen S.; Reiman D.; Clancy E.; Zielinski M.; Steinegger M.; Pacholska M.; Berghammer T.; Bodenstein S.; Silver D.; Vinyals O.; Senior A. W.; Kavukcuoglu K.; Kohli P.; Hassabis D. Highly accurate protein structure prediction with AlphaFold. Nature 2021, 596 (7873), 583–589. 10.1038/s41586-021-03819-2. 34265844 PMC8371605

[ref33] Tartaglia G. G.; Cavalli A.; Vendruscolo M. Prediction of Local Structural Stabilities of Proteins from Their Amino Acid Sequences. Structure 2007, 15 (2), 139–143. 10.1016/j.str.2006.12.007. 17292832

[ref34] Vijayakumar S.; Vishveshwara S.; Ravishanker G.; Beveridge D. L. Differential stability of beta-sheets and alpha-helices in beta-lactamase: a high temperature molecular dynamics study of unfolding intermediates. Biophys. J. 1993, 65 (6), 2304–12. 10.1016/S0006-3495(93)81288-8. 8312470 PMC1225972

[ref35] Emberly E. G.; Mukhopadhyay R.; Tang C.; Wingreen N. S. Flexibility of β-sheets: Principal component analysis of database protein structures. Proteins: Struct., Funct., Bioinf. 2004, 55 (1), 91–98. 10.1002/prot.10618. 14997543

[ref36] Emberly E. G.; Mukhopadhyay R.; Wingreen N. S.; Tang C. Flexibility of alpha-helices: results of a statistical analysis of database protein structures. J. Mol. Biol. 2003, 327 (1), 229–37. 10.1016/S0022-2836(03)00097-4. 12614621

[ref37] Parui S.; Jana B. Relative Solvent Exposure of the Alpha-Helix and Beta-Sheet in Water Determines the Initial Stages of Urea and Guanidinium Chloride-Induced Denaturation of Alpha/Beta Proteins. J. Phys. Chem. B 2019, 123 (42), 8889–8900. 10.1021/acs.jpcb.9b06859. 31574221

