# Supplementary material for: Mapping HDX-MS Data to Protein Conformations through Training Ensemble-Based Models
Source: J Am Soc Mass Spectrom. 2023 Aug 7;34(9):1989–97. doi: 10.1021/jasms.3c00145 (PMC10485923; doi:10.1021/jasms.3c00145)
Supplement: Supplementary file 1 — js3c00145_si_001.pdf [file js3c00145_si_001.pdf]

Mapping HDX-MS Data to Protein Conformations Through Training Ensemble-Based Models

Ramin E. Salmas, Matthew J. Harris and Antoni J. Borysik*

Department of Chemistry, Britannia House, King's College London SE1 1DB UK

Corresponding Author email address: antoni.borysik@kcl.ac.uk

Supporting information

Figure S1

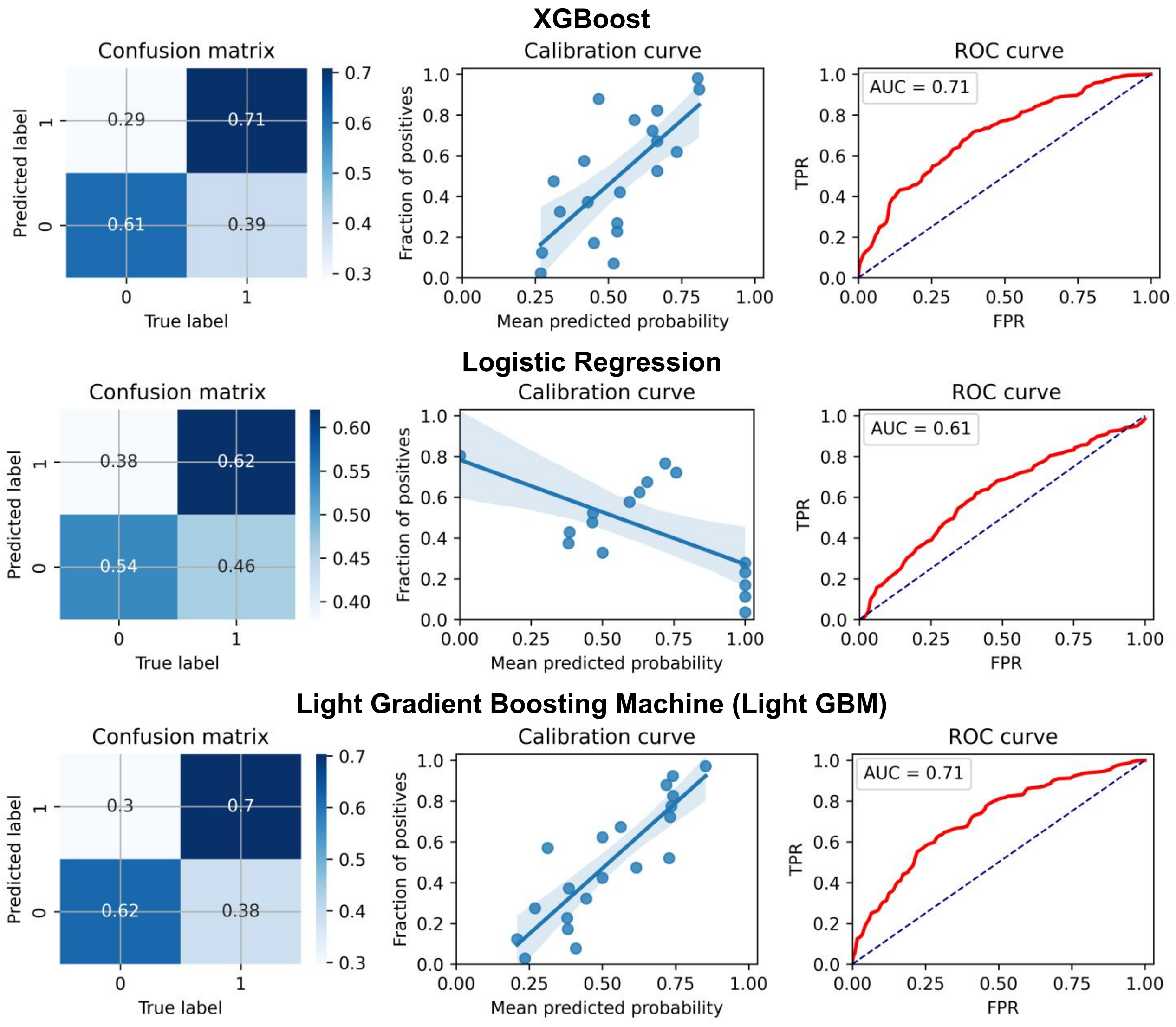

Extremely Randomised Trees

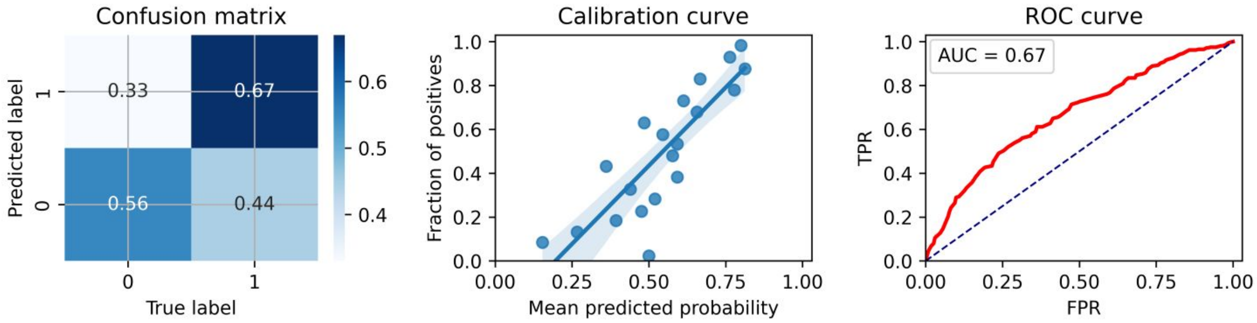

Histogram-based Gradient Boosting

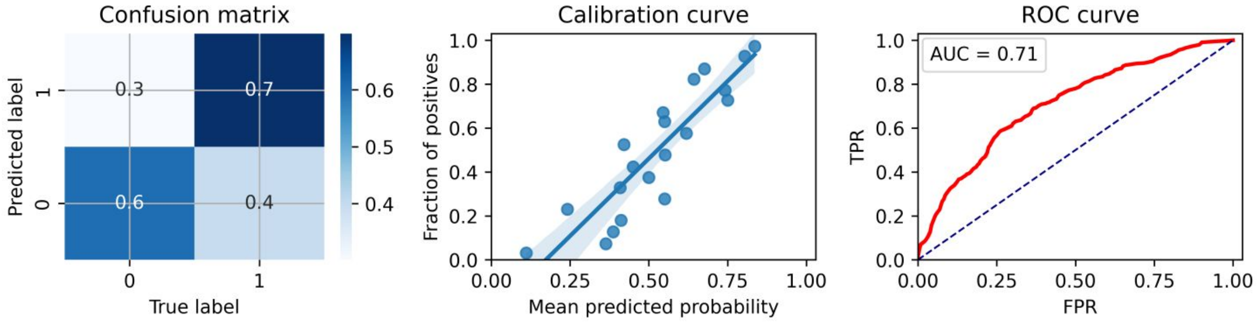

Naive Bayes

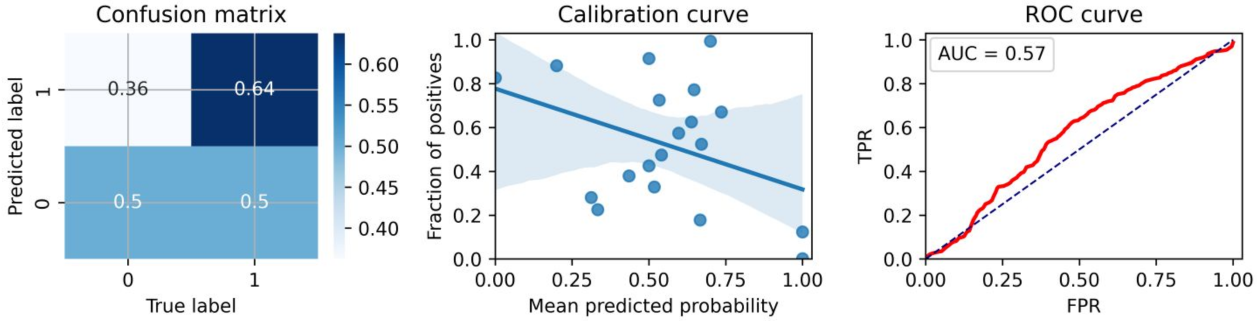

AdaBoost

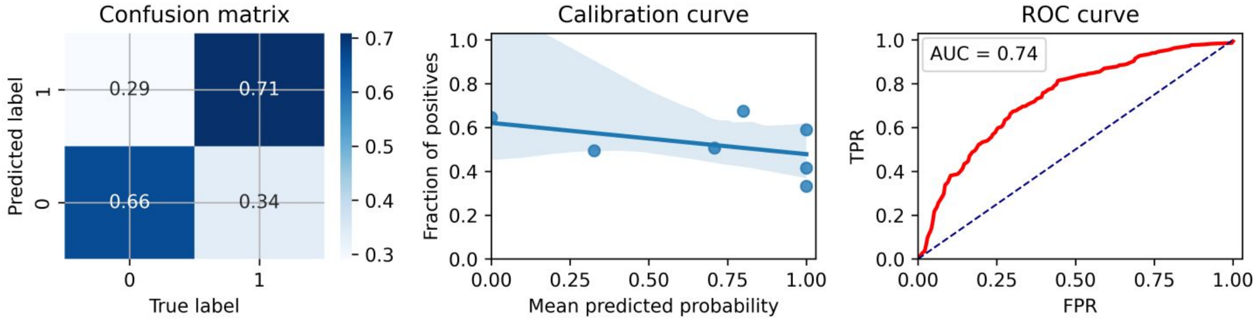

Random Forest

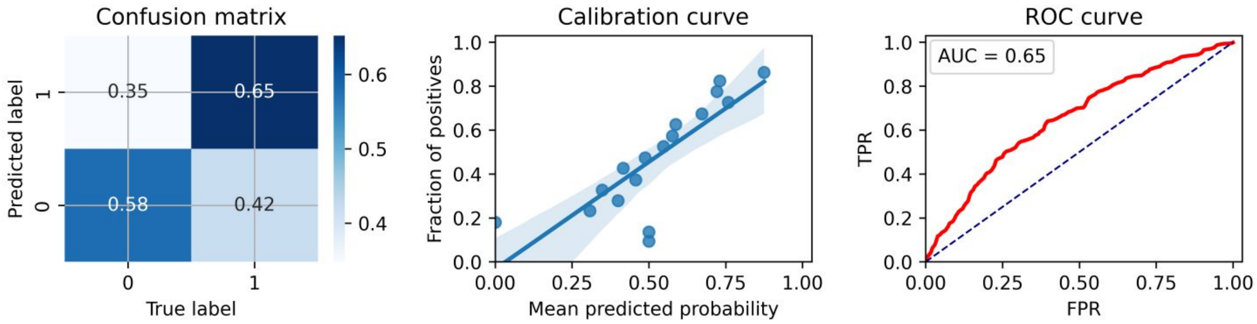

Decision Tree

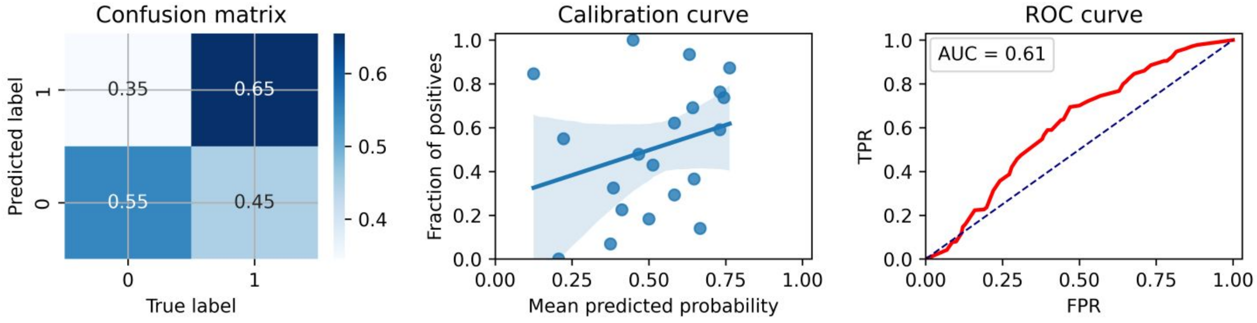

Gaussian Process

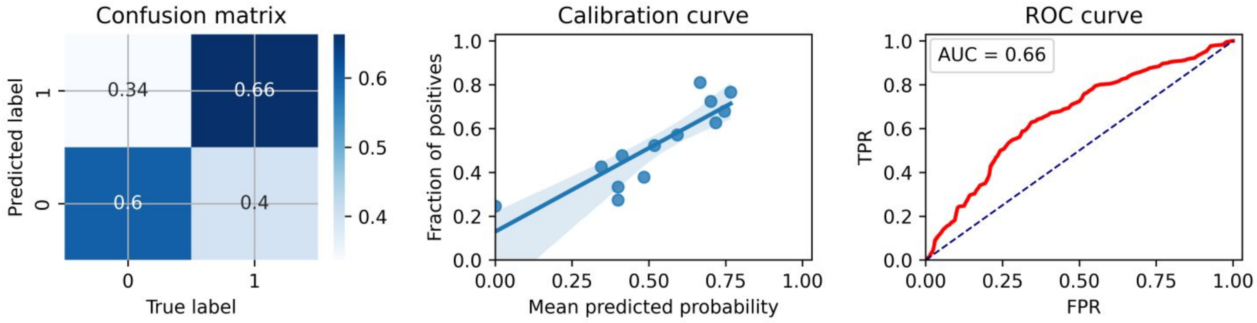

Radial Basis Function Support Vector Machines

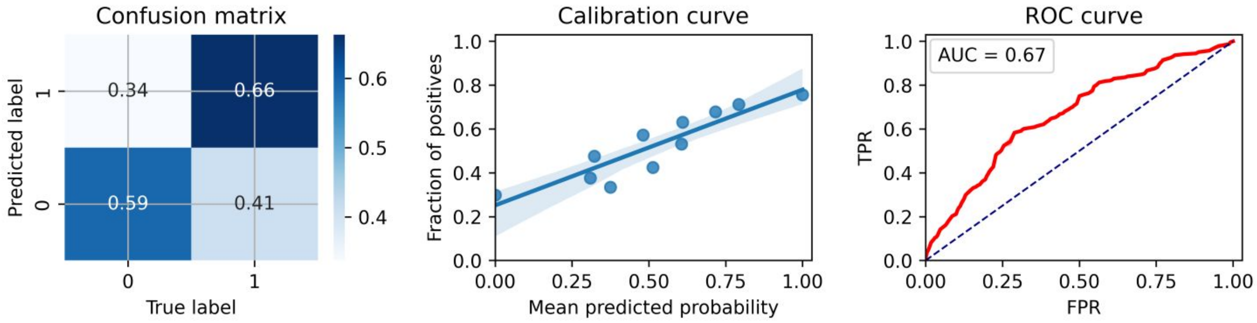

Linear Support Vector Machines

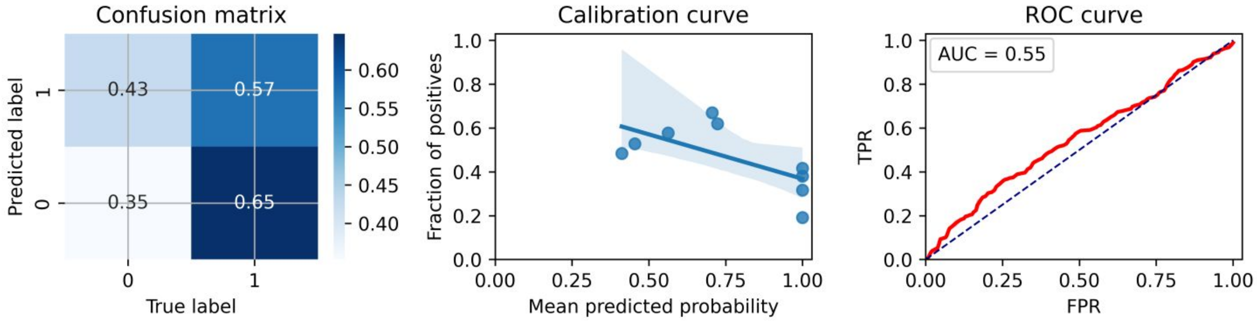

Nearest Neighbours

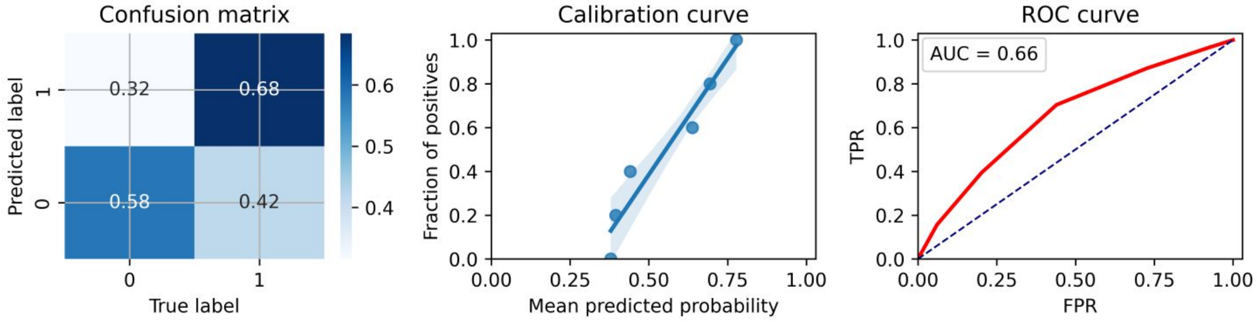

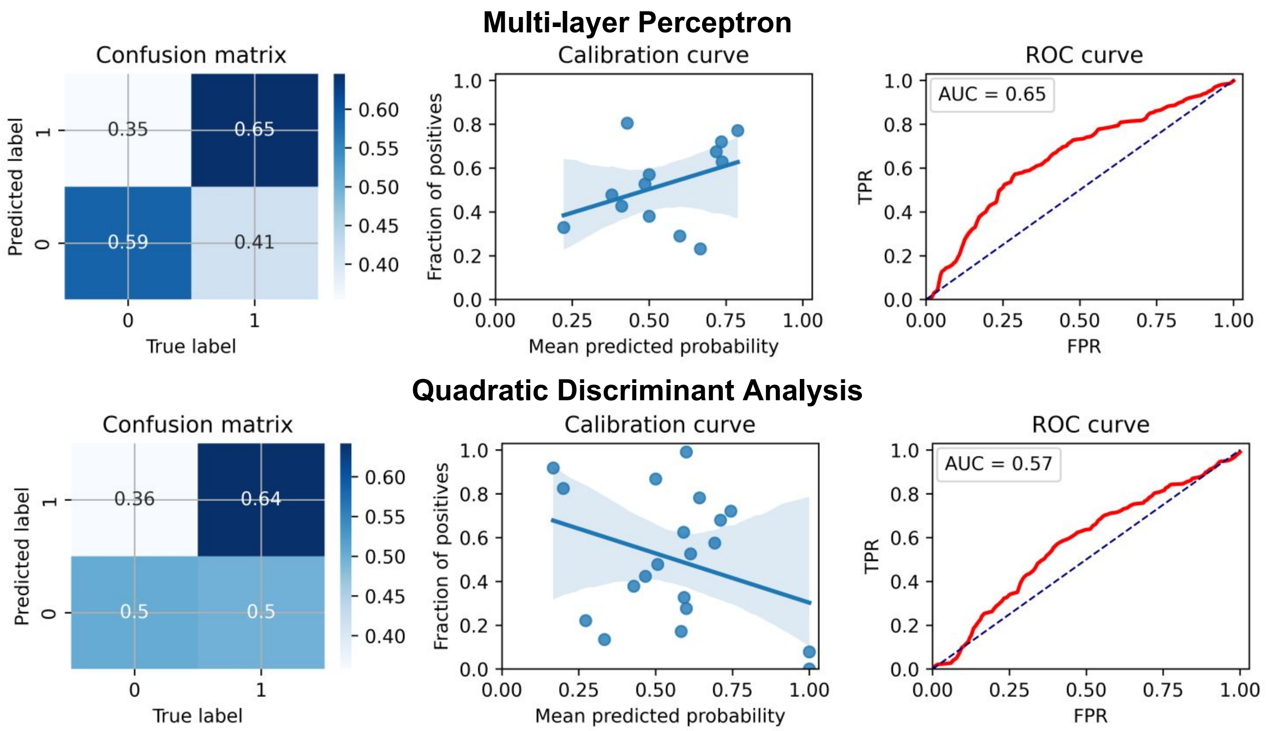

Figure S2. Ability of different types of Machine Learning algorithms to assign protein secondary structure by HDX-MS. Validation outputs are reported for 15 different types of machine learning in the form of confusion matrices, calibration curves and receiver operator characteristic (ROC) plots.
